# Diabetic Retinopathy among Diabetic Patients at a Tertiary Care Hospital: A Descriptive Cross-sectional Study

**DOI:** 10.31729/jnma.7243

**Published:** 2022-03-31

**Authors:** Pradeep Bastola, Saurav Khatiwada, Mandira Khadka, Polina Dahal, Sheeksha Bastola

**Affiliations:** 1 Department of Ophthalmology, Chitwan Medical College, Bharatpur-10, Chitwan, Nepal; 2 Department of Endocrinology, Chitwan Medical College, Bharatpur-10, Chitwan, Nepal; 3 NAME Institute for Medical Education, Kathmandu-03, Nepal

**Keywords:** *biomarkers*, *body mass index*, *diabetes mellitus*, *diabetic retinopathy*, *visual acuity*

## Abstract

**Introduction:**

The alarming rise in the prevalence of diabetes mellitus is a global public health and economic problem. Diabetic retinopathy is the most common ocular morbidity in the diabetic population and is the leading cause of blindness among the working-age group. This study was aimed to find out the prevalence of diabetic retinopathy in diabetic patients attending to the department of ophthalmology of a tertiary care hospital.

**Methods:**

A descriptive cross-sectional study was conducted in the Department of Ophthalmology of a tertiary care hospital from 15^th^ August to 30^th^ November 2021. Ethical clearance was taken from the Institutional Review Committee (Reference number: CMC-IRC/078/079-021). Convenience sampling was done. Basic demographic data, risk factors, clinical characteristics, and prevalence of diabetic retinopathy were noted. Data entry was done using Statistical Package for the Social Sciences version 26.0. Point estimate at 95% Confidence Interval was calculated along with frequency and percentage for binary data.

**Results:**

Among 540 participants, 146 (27.04%) (23.29-30.79 at 95% Confidence Interval) study subjects had diabetic retinopathy changes in at least one eye. Smoking in 88 (60.27%), type 2 diabetes mellitus in 139 (95.21%), hypertension in 85 (58.22%), raised glycated hemoglobin levels in 120 (82.19%) were the major factors observed in the study subjects.

**Conclusions:**

The prevalence of diabetic retinopathy was higher in our study when compared to national studies.

## INTRODUCTION

Diabetic retinopathy is one of the important causes of visual impairment and blindness in the world, and Nepal is not an exception to this global burden. The alarming rise in the prevalence of diabetes mellitus (DM) is a global public health problem.^1^ Diabetic retinopathy is one of the most serious complications of diabetes that imposes a great burden on the patient and the healthcare system. It involves damage to the microvasculature of the retina from prolonged exposure to the metabolic changes associated with diabetes.^2^

Visual impairment as a result of diabetic retinopathy has a significant impact on patients' quality of life, resulting in an overall negative impact on life expectancy and productivity.^3^

There is a paucity of data regarding the prevalence of diabetic retinopathy from central Nepal. This study thus primarily aimed to find out the prevalence of diabetic retinopathy in diabetic patients of central Nepal.

## METHODS

This descriptive cross-sectional study was conducted at Chitwan Medical College and Teaching Hospital (CMCTH) in the Department of Ophthalmology.

After obtaining ethical clearance from Institutional Review Committee (Reference number: CMC-IRC/078/079-021 ), the study was commenced and conducted over three- and half-month period from 15^th^ August to 30^th^ November 2021. All diabetic patients visiting the Department of Ophthalmology and referred cases from the endocrinology department of CMCTH willing to get enrolled in the study were included in the study. The patients who were not willing, who did not provide informed consent, patients with chronic debilitating systemic conditions, patients encountered in emergency wards, diabetics below 15 years of age and above 85 years of age were excluded from the study. Convenience sampling technique was used.

The sample size was calculated using the formula,

n = (Z^2^ × p × q) / e^2^

  = (1.96^2^ × 0.194 × 0.806) / 0.05^2^

  = 240

Where,

n = required sample size,
Z = 1.96 at 95% Confidence Interval (CI),
p = prevalence of diabetic retinopathy reported by a similar study, 19.4%^4^
q = 1-p
e = margin of error, 5%

The required sample size was 240, As convenience sampling was done, the sample size was doubled to 480. Considering the non-respondent rate of 10%, the total sample size becomes 528. However, our study included 540 study participants.

After explaining the purpose of the study and the confidentiality of data collection, informed consent was obtained from each participant. The study participants were evaluated in detail in the following sequence: visual acuity measurement of each eye separately (unaided and with a pinhole), extra-ocular movement assessment, cover test, cover-uncover test, refraction using a Heine Beta 200 retinoscope, anterior segment examination with a slit lamp, and detailed fundus examination. Evaluation of fundus was done by a retina specialist and consultant Ophthalmologist of the department of Ophthalmology at CMCTH. Fundus evaluation was done using direct Ophthalmoscope and indirect Ophthalmoscopy using +20 Dioptre (D), + 78D, and +90D lenses. Tropicamide 1% and or tropicamide 0.8% + phenylephrine 5% eye drops were used to dilate the pupils for fundus evaluation. All four quadrants of the retina superior, inferior, nasal, and temporal were examined in detail. The macular and foveal region was given special attention during the fundus evaluation. The significant findings from the fundus were documented and a picture of the fundus was drawn only when positive findings were observed. Modified Airlie House classification and early treatment and diabetic retinopathy study (ETDRS) were used for evaluation of different stages of diabetic retinopathy and macular edema in the study subjects.^5^

Baseline socio-demographic characteristics, awareness about diabetic retinopathy in DM patients, visual morbidity and visual status and the ocular status of the study subjects, data regarding associated factors like family history of DM, duration of DM in years, type of DM, smoking habit, alcohol consumption, body mass index, co-morbid conditions like hypertension (HTN), etc were entered in a specifically made proforma for the study. In addition, investigations like blood sugar levels, serum total cholesterol levels, glycated hemoglobin levels (HbAlc) were done in all the study subjects. The atherogenic index of plasma (AIP) was also calculated which is a known potential risk factor in the development of diabetic retinopathy.

The atherogenic index of plasma (AIP) was calculated using the following formula.^19^

AIP = Log (TG/HDL- Total Cholesterol)
AIP < 0.11 - low risk
AIP (0.11 - 0.21) intermediate risk
AIP > 0.21 increased risk

Statistical Package for the Social Sciences (SPSS) version 26.0 was used for data entry and analysis. Descriptive statistics were applied, and results were expressed as frequencies whereas continuous variables were expressed as mean±SD or median. Point estimate at 95% Confidence Interval (CI) was calculated.

## RESULTS

Out of 540 study subjects examined, 146 (27.04%) (23.29-30.79 at 95% Confidence Interval) study subjects had diabetic retinopathy at least in one eye. Mild non-proliferative diabetic retinopathy (NPDR) was the commonest diabetic retinopathy, seen in 73 (50%) of the study subjects followed by Moderate NPDR in 29 (21.23%). Clinically significant macular edema (CSME) was most prevalent in moderate non proliferative diabetic retinopathy (Moderate NPDR) (Table 1).

**Table 1:** Types of diabetic retinopathy based on severity (n = 146).

Variables	n (%)
Mild NPDR	73 (50)
Moderate NPDR	31 (21.23)
Mild to Moderate NPDR without CSME	12 (8.22)
Moderate NPDR with CSME	8 (5.48)
Severe NPDR	2 (1.37)
Severe NPDR without CSME	4 (2.74)
Severe NPDR with CSME	8 (5.48)
Early PDR	2 (1.37)
High risk PDR	2 (1.37)
High risk PDR without CSME	2 (1.37)
High risk PDR with CSME	1 (0.68)
Advanced PDR	1 (0.68)

The mean age of the study participants was 56.4±10.8 years (31-85). One hundred two (69.86%) study subjects with DR were above 50 years of age. Diabetic retinopathy was more prevalent in the female gender 77 (52.74%). The majority of the study subjects 124 (84.93%) were from urban areas. Occupation-wise, diabetic retinopathy changes were more prevalent in housewives 56 (38.36%) followed by government or non-government job holders 43 (29.45%) (Table 2).

**Table 2:** Demographic characteristics among patients with diabetic retinopathy (n= 146).

Characteristics	n (%)
**Age group**	
Below 50 Years of age	44 (30.14)
Above 50 Years of age	102 (69.86)
**Gender distribution**	
Male	69 (47.26)
Female	77 (52.74)
**Address**	
Urban	124 (84.93)
Rural	22 (15.07)
**Occupation**	
Housewife	56 (38.36)
Government/Non-government job holders	43 (29.45)
Retired	29 (19.86)
Business/Shopkeeper	18 (12.33)

Only 67 (45.89%) study participants were overweight according to body mass index (BMI). Eighty-two (56.16%) study subjects had a family history of DM. One hundred thirty-nine (95.2%) of study participants had Type 2 DM. Seventy eight (53.42%) of study participants were not aware of the development of DR in DM. Duration of diabetes mellitus was more than 10 years in 84 (57.53%) of the study subjects. Eighty-eight (60.27%) of the study subjects were smokers. Ninety-four (64.38%) study subjects did not consume any form of alcohol. Hypertension, in 85 (58.22%) study subjects was the commonest co-morbid condition. Presenting best-corrected visual acuity (VA) was normal in 116 (79.45%) study subjects (Table 3).

**Table 3:** Profile of patients with diabetic retinopathy and visual status (n= 146).

Characteristics	n (%)
**Body Mass Index**	
Normal	79 (54.11)
Overweight	67 (45.89)
**Family history of diabetes mellitus**	
Yes	82 (56.16)
No	64 (43.84)
**Type of DM**	
Type 1 DM	7 (4.79)
Type 2 DM	139 (95.21)
**Awareness regarding diabetic retinopathy**	
Not aware	78 (53.42)
Aware	68 (46.58)
**Duration of DM in years**	
More than 10 years	84 (57.53)
Less than 10 years	62 (42.47)
**Smoking habit**	
Yes	88 (60.27)
Never a smoker	58 (39.73)
**Alcohol consumption**	
No	94 (64.38)
Yes	52 (35.62)
**Co-morbid disease conditions**	
Hypertension	85 (58.22)
None	61 (41.78)
Cardiac problems	2 (1.37)
Total	146 (100)
**Best corrected visual acuity (VA)**	
Normal VA	116 (79.45)
Mild visual impairment (VI) - 6/24-6/60	23 (15.75)
Moderate VI - 5/60-3/60	5 (3.43)
Severe VI/Blind - <3/60	2 (1.37)

The mean duration of DM in the study participants was 9.1 (12.5±4.5) years. HbA1c levels in the study participants was 8.5% (5.14±2.0). HbA1c levels were raised in 120 (82.19%) study subjects. Total cholesterol level was normal in 109 (74.66%) study subjects. The atherogenic index of plasma (Log TG/HDL) was raised in 144 (98.63%) of the study subjects. Fasting blood sugar (FBS) levels and postprandial blood sugar (PPBS) levels were raised in 126 (86.30%) and 125 (85.62%) study subjects respectively. The total cholesterol levels were normal in majority of the patients (Table 4).

**Table 4:** Investigative findings in diabetic retinopathy (n = 146).

Glycated hemoglobin (HbAlc) levels	n (%)
Normal	26 (17.81)
Raised	120 (82.19)
**Total cholesterol level**	
Normal	109 (74.66)
Raised	37 (25.34)
**Atherogenic index of plasma (Log TG/HDL)**	
Increased risk	144 (98.63)
Intermediate risk	2 (1.37)
**Fasting blood sugar (FBS) level**	
Raised	126 (86.30)
Normal	20 (13.70)
**Post prandial blood sugar (PPBS) level**	
Raised	125 (85.62)
Normal	21 (14.38)

## DISCUSSION

The current study included 540 study participants; 1080 eyes of these participants were examined in detail. The prevalence of diabetic retinopathy in the current study was 146 (27.04%). Mild NPDR and moderate non-proliferative diabetic retinopathy together constituted 104 (71.2%) of study subjects with diaetic retinopathy, whereas; sight-threatening diabetic retinopathy including clinically significant macular edema (CSME) constituted 30 (20.5%) of study subjects. A number of studies show marked differences in the prevalence of retinopathy in patients with type 2 DM.^7-11^ The prevalence of diabetic retinopathy observed in our study is higher than a reported literatures^4,12^ from Nepal. These studies reported a prevalence of 19.4% and 10.1% respectively. However, the reported prevalence of diabetic retinopathy in the current study was lower than another similar study^13^ and studies done elsewhere in similar countries with lower-socioeconomic status.^14,15^ The high prevalence seen in our study could be due to the fact that the sample population was taken from the Department of Ophthalmology, where most of the patients were referred by an endocrinologist from the endocrinology clinic for visual complaints unlike some of the studies mentioned above which were done in communities or medical diabetic clinics. Different sampling techniques, sample size, and diagnostic methods may also have been contributing factors. The very low prevalence (10.1%) of diabetic retinopathy seen in the study by Thapa, et al.^12^ when compared to the current study may be due to the fact that these patients had better access to health care and in addition had good control of blood sugar and glycated hemoglobin levels.

The prevalence of sight-threatening diabetic retinopathy (Severe NPDR, CSME, Proliferative diabetic retinopathy) in the current study was 30 (20.5%). The finding of the high prevalence of sight-threatening diabetic retinopathy (STDR) in the current study did not correlate with the findings of another study^16^ which reported a lower prevalence of STDR of 15.3% and 11.9% in Type 1 DM and Type 2 DM, respectively. The higher prevalence of STDR in our study could have been attributed to the smaller study population, longer duration of diabetes mellitus in the participants, predominantly hypertensive study participants, poor glycemic control amongst the study subjects, and a large number of smokers in the study population.

The mean age of the study participants in the current study was 56.4 years (31-85 ± 10.8). The majority of the study subjects with diabetic retinopathy 102 (69.9%) were above 50 years of age. Gender wise females just outnumbered the males. The majority of the study subjects 124 (84.9%) were from urban areas. Occupation-wise, diabetic retinopathy changes were more prevalent in housewives followed by government or non-government jobs. These findings of the current study were comparable to various studies done across the globe.^13,17,20^ Females outnumbering the males in the current study was comparable to the another study.^18^ This was attributed probably to a large number of diabetic females being included in the study.

The patient-related risk factors that do develop diabetic retinopathy are well known. In the current study family history of diabetes, Type 2 DM, longer duration of DM (more than 10 years), awareness regarding diabetic retinopathy in the DM patients (78, 53.4% - not aware), concurrent co-morbid conditions like hypertension and smoking habits (Table 3) were predominant in study subjects with diabetic retinopathy. These findings from the study were comparable to studies done elsewhere.^17-20^ Inclusion of more patients with type 2 DM in the current study meant that it was a risk factor to develop diabetic retinopathy later which was attributed to the persistent presence of risk factors and uncontrolled blood sugar levels in the study population.

Hypertension has been documented as a risk factor in studies from Middle East^9^ and also by a longitudinal UK prospective diabetes study group.^21^ Report from UAE reveals that diabetic retinopathy is marginally significantly associated with hypertension.^22^ While in contrast, many studies including those from Hassa (KSA), Riyadh (KSA), and from South India^23^ were not able to find any significant role of hypertension in the development of diabetic retinopathy. In our study, hypertension was the only observed prevalent co-morbid condition which is a known risk factor for diabetic retinopathy, this finding from the study was comparable with the study from the UK and the Middle East.^9,21^

BMI and smoking did not appear as significant risk factors according to studies in UAE,^22^ and Tehran;^24^ the latter finding is in contrast to a report from Madina,^25^ and South India^23^ that documented smoking to be associated with an increased prospect of diabetic retinopathy, which correlated very well with our study findings. Eighty-eight (60.3%) study subjects with diabetic retinopathy were smokers in the current study.

The ocular and visual status of the study participants were predominantly normal in our current study (Table 3) as non-sight threatening diabetic retinopathy was more prevalent in the current study.

Poor glycemic control is a risk factor for the development and progression of diabetic retinopathy and is associated with a higher prevalence of diabetic retinopathy as shown by reports from different studies. Glycated hemoglobin level (HbA1c) is one of the most reliable indicators for glycemic control over a period of time.^13^ In the current study HbA1c levels, fasting blood sugar, and postprandial sugar levels were analyzed to find out glycemic control in the study population. As the study was conducted in a tertiary level hospital, and with co-morbid conditions like hypertension being more prevalent in the study population, total cholesterol level, triglycerides, high-density lipoprotein, and low-density lipoprotein levels were also analyzed to ensure better treatment modalities for the study subjects. The atherogenic index of plasma was used as a serum biomarker for predictive risk for future development of cardiac events, and to observe the development of diabetic retinopathy.

In the present study, glycated hemoglobin level (HbA1c), fasting blood sugar (FBS), post-prandial blood sugar (PPBS) was raised predominantly in patients with diabetic retinopathy. These findings from the study correlated and compared very well with the current understanding of the literature on the development of diabetic retinopathy in people with poor glycemic control over a period of time.^13-16^ The findings from the current study further established the fact that poor glycemic control is one of the key risk factors responsible for progress to retinopathy changes in diabetic patients.

The total cholesterol levels were normal in 109 (74.7%) study subjects in the current study. Study has been reported which did not find any significant association of serum lipids with development of diabetic retinopathy in patients with diabetes.^26^ Atherogenic dyslipidemia is characterized by low high-density lipoprotein cholesterol (HDL-c) and/or high triglyceride levels. This dyslipidemia is particularly common in type 2 DM and has been reported to be associated with both microangiopathy and residual cardiovascular risk in type 2 DM patients.^27-29^ In the current study we analyzed the atherogenic index of plasma in the study subjects and found it was significantly raised and was contributing to the development of diabetic retinopathy.

The findings from our study were comparable to various studies across the globe and were not comparable to some studies done in Nepal and abroad. We would like to further recommend exploring risk factors associated with the development of diabetic retinopathy and diabetes mellitus particularly focusing on atherogenic dyslipidemia as a serum biomarker and conducting similar studies from other parts of the country.

## CONCLUSIONS

The prevalence of diabetic retinopathy in the current study was higher than the national estimates and lower or similar to the reported prevalence from international literature. Mild non-proliferative diabetic retinopathy (NPDR) and moderate NPDR were the most common types of diabetic retinopathy observed. Non-sight threatening diabetic retinopathy was predominant in the study. Longer duration of diabetes mellitus, smoking, concurrent co-morbid conditions like hypertension, poor glycemic control, raised atherogenic index of plasma were the predominant risk factors observed in these patients with diabetic retinopathy. Continuous effort is required from healthcare professionals in counseling diabetic patients about the role of blood sugar level and hypertension control in reducing the risk of onset and progression of diabetic retinopathy. Health education for diabetic patients about the need to have regular eye evaluation for early detection and management of diabetes-related eye complications is recommended through awareness campaigns like World Diabetes Day.
